# Machine learning applications in the analysis of sedentary behavior and associated health risks

**DOI:** 10.3389/frai.2025.1538807

**Published:** 2025-06-18

**Authors:** Ayat S Hammad, Ali Tajammul, Ismail Dergaa, Maha Al-Asmakh

**Affiliations:** ^1^Department of Biomedical Sciences, College of Health Sciences, QU Health, Qatar University, Doha, Qatar; ^2^Biomedical Research Center, Qatar University, Doha, Qatar; ^3^High Institute of Sport and Physical Education of Ksar Saïd, University of Manouba, Manouba, Tunisia; ^4^Physical Activity Research Unit, Sport and Health (UR18JS01), National Observatory of Sports, Tunis, Tunisia; ^5^High Institute of Sport and Physical Education of El Kef, University of Jendouba, Jendouba, Tunisia

**Keywords:** health risks, inactivity, lifestyle disease, machine learning, modeling, physical activity, sedentary behavior, wearables

## Abstract

**Background:**

The rapid advancement of technology has brought numerous benefits to public health but has also contributed to a rise in sedentary lifestyles, linked to various health issues. As prolonged inactivity becomes a growing public health concern, researchers are increasingly utilizing machine learning (ML) techniques to examine and understand these patterns. ML offers powerful tools for analyzing large datasets and identifying trends in physical activity and inactivity, generating insights that can support effective interventions.

**Objectives:**

This review aims to: (i) examine the role of ML in analyzing sedentary patterns, (ii) explore how different ML techniques can be optimized to improve the accuracy of predicting sedentary behavior, and (iii) assess strategies to enhance the effectiveness of ML algorithms.

**Methods:**

A comprehensive search was conducted in PubMed and Scopus, targeting peer-reviewed articles published between 2004 and 2024. The search included the subject terms “sedentary behavior,” “sedentary lifestyle health,” and “machine learning sedentary lifestyle,” combined with the keywords “physical inactivity” and “diseases” using Boolean operators (AND, OR). Articles were included if they addressed the health impacts of sedentary behavior or employed ML techniques for its analysis. Exclusion criteria involved studies older than 20 years or lacking direct relevance. After screening 33 core articles and identifying 13 more through citation tracking, 46 articles were included in the final review.

**Results:**

This narrative review describes the characteristics of sedentary behavior, associated health risks, and the applications of ML in this context. Based on the reviewed literature, sedentary behavior was consistently associated with cardiovascular disease, metabolic disorders, and mental health conditions. The review highlights the utility of various ML approaches in classifying activity levels and significantly improving the prediction of sedentary behavior, offering a promising approach to address this widespread health issue.

**Conclusion:**

ML algorithms, including supervised and unsupervised models, show great potential in accurately detecting and predicting sedentary behavior. When integrated with wearable sensor data and validated in real-world settings, these models can enhance the scalability and precision of AI-driven interventions. Such advancements support personalized health strategies and could help lower healthcare costs linked to physical inactivity, ultimately improving public health outcomes.

## 1 Introduction

A sedentary lifestyle, characterized by participation in low-energy activities such as watching television, sitting, or using electronic devices, with energy expenditures of ≤1.5 METs (metabolic equivalents), is recognized as a significant global health concern (Teychenne et al., 2015; Liu et al., 2016). The physical inactivity associated with modern lifestyles has been linked to an increased risk of developing cardiovascular diseases, including type 2 diabetes, strokes, and heart attacks, as well as cognitive disorders like dementia and cancers such as colorectal and breast cancer (WHO, 2024). Conversely, regular physical activity has been shown to improve functional capacity, reduce the risk of chronic diseases, support weight management, and provide psychological benefits, including reduced anxiety and depression and improved mood (An et al., 2020). As a result, health professionals emphasize the importance of increasing physical activity levels and reducing prolonged inactivity behavior (Katzmarzyk, 2010). Light physical activity is typically characterized by energy expenditures of 1.6–2.9 METs, moderate activity by 3–5.9 METs, and strenuous activity by 6 METs or greater. These thresholds serve as guidelines for promoting healthier, more active lifestyles (Newton et al., 2013).

According to the World Health Organization (WHO), approximately 31% of adults worldwide (1.8 billion people) did not meet the recommended physical activity levels in 2022, and this figure is projected to rise to 35% of the global population by 2030 (WHO, 2024). Several factors contribute to low participation in physical activity, including environmental barriers such as air pollution, traffic congestion, limited access to sports or recreational facilities, and a lack of parks and pedestrian walkways (World Health Organization, 2020). Furthermore, excessive screen time, including video viewing, television watching, and cell phone usage, further exacerbates the issue (Fennell et al., 2019). The rapid advancement of technology over recent years has significantly reduced physical activity, contributing to negative health outcomes. As a result, the relationship between physical activity and health has become a central focus of research (Hamilton et al., 2008).

Over the years, the application of machine learning (ML) has played an important role in investigating several research problems. ML is a data-driven approach designed for problem-solving, particularly adept at managing complex and high-dimensional datasets (Bellazzi et al., 2011; Rajpurkar et al., 2022; Wiemken and Kelley, 2020; Haug and Drazen, 2023). As a subfield of artificial intelligence, ML encompasses a diverse set of methodologies aimed at recognizing patterns and learning from data (Haug and Drazen, 2023; Javaid et al., 2022). It focuses primarily on pattern recognition and predictive modeling, enabling the extraction of insights and predictions from vast and intricate datasets (Haug and Drazen, 2023; Javaid et al., 2022). This makes ML a valuable asset in the study of sleep behavior, physical activity, and sedentary lifestyle patterns, where it can uncover nuanced relationships and provide evidence-based insights (Farrahi and Rostami, 2024).

In the context of physical activity, ML techniques have been predominantly employed to estimate energy expenditure, quantify movement intensity, and classify activity types using data derived from wearable devices (Farrahi et al., 2019; Freedson et al., 2012; Narayanan et al., 2020). For example, accelerometers worn on the thigh and hip have demonstrated high accuracy in identifying sit-to-stand transitions and detecting sitting patterns, providing detailed insights into sedentary behavior and postural transitions (Greenwood-Hickman et al., 2021). These capabilities underscore the utility of ML in analyzing complex movement data, offering precise assessments of physical activity and related health outcomes. Additionally, several studies have utilized decision trees, ML algorithms, and random forest techniques to automatically sift through extensive sets of potential predictors, identifying the most influential factors related to physical activity and sedentary behaviors (Biswas et al., 2023; Cheng et al., 2020; Buck et al., 2019). This approach enhances the ability to pinpoint critical variables, facilitating targeted interventions and advancing our understanding of behavioral patterns linked to health.

Since sedentary behavior may lead to different diseases, it is important to classify different activities accurately, which requires further research as that can aid in assessing and predicting sedentary behaviors. Although machine learning has played a pivotal role in the research on sedentary lifestyle using approaches based on steps counting and analyzing wearable sensors, the algorithms have not been often tested in completely free-living conditions. Hence, there is a need to test the machine learning algorithms under completely free-living conditions.

The purpose of this review was (i) to explore the understanding of a sedentary lifestyle and its health implications, (ii) to investigate the effectiveness of machine learning approaches in studying sedentary behaviors, and (iii) to access the measures required to further improve the effectiveness of machine learning algorithms.

## 2 Materials and methods

The search strategy involved a comprehensive database search of PubMed and Scopus using the subject terms “sedentary behavior,” “sedentary lifestyle health,” and “machine learning sedentary lifestyle.” These databases were selected because they primarily include articles from peer-reviewed, high-quality journals that adhere to the highest academic standards. Only the publications of the last 20 years (from 2004 to 2024) were included.

To accomplish the goal of researching the impact of sedentary lifestyle on health, the subject terms “sedentary behavior” and “sedentary lifestyle health” were combined with the keywords “physical inactivity” and “diseases” using Boolean operators (AND, OR). Then, to explore the role of machine learning in studying sedentary lifestyle, the subject term used was “machine learning sedentary lifestyle”; it was combined with the keyword “physical inactivity” with the Boolean operators (AND, OR). To explore the role of machine learning techniques in analyzing sedentary lifestyle, emphasis was placed on articles with case studies.

The exclusion criterion was that the article should not be over 20 years old. The studies used in the review were based on the relevance with regards to the health effects of a sedentary lifestyle and the use of different machine learning approaches in studying prolonged inactivity lifestyles; ergo, any study not specifically constituting that was not included in the review.

From the initial database search, a total of 33 articles were initially identified from PubMed and Scopus using defined search terms and Boolean logic. After applying inclusion/exclusion criteria (peer-reviewed, post-2004, direct relevance to sedentary behavior and ML), we screened abstracts and full texts. An additional 13 articles were included through citation tracking, bringing the total to 46. Screening was done independently by two reviewers.

## 3 Understanding sedentary lifestyle

A sedentary lifestyle involves behaviors constituting low levels of energy expenditure defined by metabolic equivalent task (MET) values, defined as a multiple of resting energy expenditure (REE) and typically represented as 3.5 mL/kg/min (Farrahi and Rostami, 2024; Saint-Maurice et al., 2016). Typically, physical activity is defined based on MET values with 1.5- < 3 METs for light exercise, 3- < 6 METs for moderate activity and >6 METs for vigorous activity (Saint-Maurice et al., 2016). Sedentary behavior can be generally defined as sitting or reclining with the energy-expenditure range of around 1.0 to 1.5 METs (Owen, 2012). Some examples of sedentary behavior include watching television, playing video games, using a computer, and sitting at school/work or while commuting. As societies have modernized and technology has advanced, the reliance on technology has escalated resulting in high sedentary behavior engagement (Waters et al., 2016). This prevalence of prolonged inactivity lifestyle globally poses a big health risk.

## 4 Health implications of sedentary lifestyles

The understanding of inactivity-driven lifestyle in the context of disease holds significance in investigating the impact of physical activity on health and in designing relevant interventions. Prolonged times of sedentary behavior are associated with poor disease outcomes, and it can lead to several diseases (Park et al., 2020).

### 4.1 Cardiovascular diseases

Sedentary lifestyle is associated with cardiovascular diseases and premature mortality (Park et al., 2020). Three articles in our review highlighted the association between cardiovascular diseases and sedentary lifestyle. One study revealed that the risk of all-cause mortality increases as the total daily sedentary time and television viewing time increase (Katzmarzyk et al., 2019). The detrimental impact of inactivity-driven lifestyle behavior was found to be more evident among people involved in little physical activity relative to those doing frequent physical activity (Biswas et al., 2015). The relative risk for all-cause mortality was 30% higher in individuals with high physical activity as compared to individuals with low physical activity (Biswas et al., 2015).

### 4.2 Metabolic disorders

Sedentary lifestyle has been found to significantly link with metabolic diseases (Park et al., 2020). Seven articles in our review have linked metabolic disorders with sedentary behaviors. The prevalence of type 2 diabetes mellitus increases as the sedentary time increases (Biswas et al., 2015). In that, the level of physical activity does not influence the effect of prolonged sedentary time on the risk for diabetes mellitus. With that, the problem of hypertension (high blood pressure) rises with increase in sedentary time. Prolonged sedentary time reduces the metabolic demands and systemic blood flow while increasing oxidative stress and promoting low-grade inflammatory cascade (Dempsey et al., 2018); hypertension is a significant risk factor for cardiovascular disease. Additionally, sedentary lifestyle increases the risk of metabolic dysfunction signified by increases blood triglyceride levels, reduced HDL-cholesterol levels, and diminished insulin sensitivity (Hamilton et al., 2007). A study found that the risk for dyslipidemia, imbalance of lipids like triglycerides and cholesterol, increases in both men and women due to sedentary behaviors (Zhou et al., 2017). In terms of metabolic diseases, sedentary lifestyle significantly affects obesity as well (Park et al., 2020). One study revealed that the waist circumference increased by 3.1 cm as the sedentary time increased by 10% (Healy et al., 2008). Another study found that the cause of weight gain is prolonged sedentary time (Ohlsson et al., 2020).

### 4.3 Cancer risks

Sedentary lifestyle also connects with the prevalence of cancer (Park et al., 2020). Six articles in our review have correlated cancer with sedentary lifestyle. One study found that the cancer risk in the group with the longest sedentary time was 13% higher relative to the group with the shortest sedentary time (Biswas et al., 2015). Sitting for longer periods of time increases cancer risks, including colorectal, ovarian and prostate cancers; it has also been found to increase cancer mortality, especially in women (Lynch, 2010). Television viewing time has also been reported to increase the risk of colon cancer and endometrial cancer (Schmid and Leitzmann, 2014). A plausible explanation of sedentary behavior correlating with hormone-related cancers like breast and endometrial cancers is that sedentary behavior results in metabolic dysfunctions, which includes changes in the circulation levels of sex hormones; that alteration might lead to hormone-related cancers (Tworoger et al., 2007). Additionally, as mentioned earlier, sedentary lifestyle contributes significantly to obesity which, in turn, is a risk factor for several cancers (Jochem et al., 2019).

### 4.4 Osteoporosis

Sedentary behavior is also positively associated with osteoporosis, a bone disease that occurs when bone mineral density and bone mass decrease (Park et al., 2020). There are less studies investigating the association between osteoporosis and sedentary behavior; our review has included two such articles. One study found that the bone mineral density of the total femur correlated negatively with the sedentary time in adult women (Chastin et al., 2014). The duration (and not the frequency) of sedentary behavior was shown to impact bone mineral density; sedentary behavior did not show significant correlation with bone mineral density of the hip and spine in men (Chastin et al., 2014).

### 4.5 Musculoskeletal issues

Sedentary lifestyle has been linked with musculoskeletal diseases too (Park et al., 2020). Two articles included in this review have highlighted the link of musculoskeletal diseases with sedentary lifestyle. One study revealed that the incidence of chronic knee pain was higher in individuals with longer sedentary times (Lee et al., 2019). In cases where sedentary time was greater than 10 h a day was significantly correlated with chronic knee pain; based on that, the study has recommended individuals to keep their sedentary times less than 10 h a day (Lee et al., 2019).

### 4.6 Mental health

Sedentary behavior is also associated with depression (Park et al., 2020). Two articles in our review have shown the correlation between sedentary behaviors and depression. The articles have divided sedentary behaviors into two categories: mentally passive and mentally active. The mentally passive sedentary behaviors that include television viewing, listening to music, sitting and talking while sitting were shown to have a positive correlation with depression risks; on the other hand, mentally active sedentary behaviors like reading books or newspapers, driving, attending a meeting were shown to not have a significant correlation with depression risk (Huang et al., 2020). One possible explanation of this trend in the correlation is that the sedentary behaviors may increase the risk for depression by blocking direct communication and reducing social interactions, or by decreasing the time that could be spent in physical activities which aid in preventing depression (Huang et al., 2020).

### 4.7 Overview of health risks of sedentary lifestyle

It can be observed that sedentary behaviors can drastically impact the health of individuals which calls for more research on this lifestyle upon which relevant interventions can be developed to mitigate its impact. Over the years, machine learning has played a significant role in classifying activities and in accurately predicting sedentary behaviors. Sedentary behavior has been linked to numerous health risks, including cardiovascular diseases, metabolic disorders, and mental health conditions. Table 1 summarizes these health implications, highlighting the association between sedentary behavior and various disease outcomes.

**Table 1:** The health implications of sedentary behavior.

Disease	Impact of sedentary behavior	Studies
Cardiovascular	Chances of all-cause mortality increase	Katzmarzyk et al. (2019)
Metabolic	Risk of type 2 diabetes mellitus, hypertension, dyslipidemia and obesity increase	Biswas et al. (2015), Dempsey et al. (2018), Zhou et al. (2017), and Healy et al. (2008)
Cancer	Cancer risk increases, especially for hormone-related cancers. Since obesity is impacted, that also contributes to several cancers	Tworoger et al. (2007) and Jochem et al. (2019)
Osteoporosis	Duration of sedentary behavior can impact bone mineral density	Chastin et al. (2014)
Musculoskeletal	Longer sedentary times can lead to musculoskeletal diseases	Lee et al. (2019)
Mental health	Mentally passive sedentary behaviors have a positive correlation with depression risks	Huang et al. (2020)

## 5 Machine learning methodologies

There are different machine learning techniques that have been used for the classification of activities. In this review, we will be briefly covering 11 different classification techniques.

### 5.1 Threshold-based classification

#### 5.1.1 Mechanism

Threshold-based classification uses predefined numerical thresholds applied to derived features (e.g., angles, acceleration values) to categorize human activity (Preece et al., 2009; Najafi et al., 2003). A threshold is set in advance, and sensor-derived values are compared against it to classify posture or motion. For example, accelerometer data can be used to distinguish between static postures such as standing, sitting, and lying based on the orientation angles of the body (Boyle et al., 2006; Coley et al., 2005; Culhane et al., 2004).

#### 5.1.2 Application and limitations

This technique has been widely adopted for sedentary behavior analysis. It has shown notable success in fall detection, despite the fact that falls represent extreme postural changes (Boyle et al., 2006). One commonly used feature is the rapid deceleration point upon ground contact, which marks the moment of fall (Chen et al., 2005). Studies have reported improved detection accuracy when combining multiple threshold rules (Chen et al., 2005; Lindemann et al., 2005; Hwang et al., 2004; Bourke and Lyons, 2008). However, the method has limitations. It is highly sensitive to sensor placement and device orientation, which may affect classification reliability. Furthermore, the approach often requires manual calibration for each user, making it less adaptable to heterogeneous populations. Lastly, threshold-based models struggle with recognizing complex transitions or dynamic sequences of movement.

### 5.2 Hierarchical methods

#### 5.2.1 Mechanism

Hierarchical classification structures decisions into a series of binary steps arranged in a tree-like architecture, where each node makes a specific classification based on selected features (Preece et al., 2009). These decision nodes are typically handcrafted and reflect domain knowledge, often requiring careful tuning through manual inspection of training data (Preece et al., 2009). The hierarchical model divides the classification task into simpler subproblems, allowing for stepwise refinement of predictions. This approach has been used to handle complex activity recognition tasks by structuring classifiers first to detect general activity classes, followed by more detailed differentiation in subsequent layers.

#### 5.2.2 Application and limitations

This approach has been used to distinguish between eight different dynamic activities by applying a threshold-based hierarchical classification scheme (Parkka et al., 2006). Karantonis et al. (2006) proposed using embedded intelligence in this approach to make it computationally efficient and simplified, with a high potential of accurately detecting real-time falls. Although this layered structure enhances modularity and real-time deployment, it also introduces limitations such as error propagation through the tree and high reliance on expert-defined rules. These constraints reduce scalability and make it difficult to adapt the system to different sensors or demographic groups.

### 5.3 Decision trees

#### 5.3.1 Mechanism

Decision tree algorithms function by recursively splitting a dataset into branches based on the discriminative power of selected features, ultimately forming a tree-like structure of rules that lead to classification outcomes (Webb, 2002; Duda et al., 2000). At each node, the algorithm evaluates which feature provides the best separation of activity types, continuing the process until terminal nodes (leaves) represent a final classification label. This method is intuitive and interpretable, allowing researchers to visualize the flow of decision logic and understand how the model reaches its conclusions.

#### 5.3.2 Application and limitations

In sedentary behavior research, decision trees have been used to distinguish among a wide variety of activities using both time- and frequency-domain features. One study achieved 86% classification accuracy across 20 different activities using five sensors (Bao and Intille, 2004). Another investigation focused on six daily activities and demonstrated that using time-domain features alone reduced computational complexity while maintaining strong performance (Maurer et al., 2006). Although decision trees are efficient and easy to implement, they are prone to overfitting, particularly when working with noisy or high-dimensional sensor data. Additionally, their performance can degrade when the training dataset lacks representative variation, limiting generalizability across different users and conditions.

### 5.4 k-nearest neighbor

#### 5.4.1 Mechanism

The k-nearest neighbor (kNN) algorithm classifies data points based on their proximity to other labeled instances within a multi-dimensional feature space (Duda et al., 2000; Theodoridis and Koutroumbas, 2006). Each axis in this space represents a different sensor-derived feature, and the training data collectively define the landscape (Bussmann et al., 2001). When a new data point is introduced, the algorithm identifies the k most similar points—its “neighbors”—and assigns the majority class label among them to the unknown point. This method is non-parametric and instance-based, meaning it makes no assumptions about the data distribution and performs classification only at query time.

#### 5.4.2 Application and limitations

In the context of sedentary behavior, kNN has been applied to detect falls by distinguishing them from normal daily movements. One study demonstrated the feasibility of using kNN for reliable and efficient fall detection, showing its potential utility for monitoring elderly populations where timely fall response is critical (Zhang et al., 2006). However, kNN’s reliance on distance calculations makes it computationally expensive with large datasets, and its performance can be sensitive to feature scaling and irrelevant dimensions. Moreover, it lacks an internal model, making it less interpretable and slower compared to tree-based methods during prediction.

### 5.5 Artificial neural networks

#### 5.5.1 Mechanism

Artificial neural networks (ANN) represent complicated relationships between the inputs and outputs, independent and dependent variables, respectively (Preece et al., 2009). Training data is employed for the optimization process so that known outputs can be predicted for a given set of inputs; an ANN can then be used to obtain outputs for any set of inputs (Preece et al., 2009). The inputs are the different features, while the outputs are the different classes of activities (Aminian et al., 1995; Goulermas et al., 2008; Goulermas et al., 2005). There are different classes of ANNs aimed at improving the classification accuracy (Preece et al., 2009).

#### 5.5.2 Application and limitations

One study drew a comparison between pulsed neural networks (PNN), which uses inputs from tilt switches, and an approach using standard accelerometer data and a self-organizing map; the researchers concluded that even though tilt switches did not outperform the approach using accelerometer data, the accuracy of the approach was still good for different daily activities (Laerhoven and Gellersen, 2004). Nevertheless, ANNs require large, labeled datasets for training and are computationally intensive, often necessitating the use of external servers or cloud processing. Additionally, their black-box nature makes them difficult to interpret, which can be a concern in clinical settings where transparency and model explainability are critical.

### 5.6 Support vector machines

#### 5.6.1 Mechanism

Support vector machines (SVMs) are supervised learning models that construct optimal decision boundaries—called hyperplanes—between classes in a feature space, maximizing the margin between the closest data points of each class (Cristianini and Shawe-Taylor, 2001; Vapnik, 1998). The algorithm can project the original feature space into a higher-dimensional space using kernel functions, allowing for more complex, non-linear separations. This optimization-based approach enables precise classification even in high-dimensional settings, making SVMs particularly powerful for activity recognition tasks that involve subtle distinctions in movement.

#### 5.6.2 Application and limitations

Not many studies have employed SVMs, although the few studies that have applied have shown great promise. The most comprehensive study by Zhang et al. (2006) involved the collection of data from a tri-axial accelerometer embedded in a cellphone placed in the pocket of clothes or hung on the neck of individuals; the results revealed that this method can detect falls effectively with an accuracy of 92.4% for activities constituting critical movement and 84.4% for high-intensity daily activities (Zhang et al., 2005). However, SVMs require careful tuning of hyperparameters and kernel functions, and their performance may degrade when working with overlapping classes or limited training data, which are common challenges in real-world wearable sensor datasets.

### 5.7 Naive Bayes and Gaussian mixture models

#### 5.7.1 Mechanism

The Bayesian approach to classification estimates the posterior probability of an activity class given the observed data, based on the likelihood of signal patterns and the prior distribution of each class (Preece et al., 2009). Naïve Bayes simplifies this process by assuming that all input features are conditionally independent, allowing the model to compute class probabilities as the product of individual feature likelihoods. A more generalized form, such as discriminant analysis, accounts for cross-feature correlations, while Gaussian mixture models (GMMs) extend this by modeling each activity’s feature distribution as a mixture of multiple Gaussian components with unknown parameters (Preece et al., 2009; Duda et al., 2000; Theodoridis and Koutroumbas, 2006).

#### 5.7.2 Application and limitations

In activity classification tasks, GMMs have been used to model time-domain features for multiple movements. One study applied subject-specific GMM training and selected the model with the highest likelihood to classify new data (Allen et al., 2006). This method proved efficient when adapted to individual users. Naïve Bayes and GMMs are computationally lightweight and easy to implement, making them attractive for embedded systems. However, the independence assumption in Naïve Bayes often fails in practical sensor data, limiting its accuracy, while GMMs require a good estimate of the number and shape of the Gaussian components, which may vary widely across individuals and behaviors.

### 5.8 Fuzzy logic

#### 5.8.1 Mechanism

Fuzzy logic, derived from fuzzy set theory, enables reasoning with imprecise or uncertain data—ideal for human activity recognition where exact thresholds may not always apply (Preece et al., 2009). In this system, input features from body-worn sensors are assigned degrees of membership to activity classes through membership functions that range between 0 and 1. These fuzzy memberships are then evaluated through if–then rules to produce a degree of confidence, or “fuzzy truth,” for each potential class. The activity with the highest fuzzy truth is typically chosen as the final classification.

#### 5.8.2 Application and limitations

Fuzzy logic has shown unique promise in sedentary behavior analysis due to its flexibility in handling ambiguous input, such as subtle postural shifts or overlapping movement patterns. Its tolerance for uncertainty makes it particularly suitable for real-world applications, where sensor data is noisy and incomplete (Preece et al., 2009). However, fuzzy systems rely on hand-crafted rule sets and membership functions, which require expert domain knowledge and limit scalability. Tuning these rules for diverse populations or varying sensor placements remains a challenge.

### 5.9 Markov chains and hidden Markov models

#### 5.9.1 Mechanism

Markov chain holds distinction in the fact that it can be used to represent the likelihood of transitions between different activities; each activity is represented as a different state (Preece et al., 2009). However, in the case of hidden Markov model (HMM), the state of the model at a given time is not known and only observable parameters depending on the state can determine that; HMM can be used directly for activity classification problems (Preece et al., 2009; Pober et al., 2006; Ward et al., 2006; Lester et al., 2006; Lester et al., 2005). The observable parameters constitute features obtained from the body-sensor data, in which the states represent different activities (Preece et al., 2009). Another factor distinguishing HMM from Markov chains is that states in HMM can correspond to more than one activity (Preece et al., 2009; Pober et al., 2006; Ward et al., 2006; Lester et al., 2006; Lester et al., 2005). The example data is used to train HMM, which can then be used to establish the most likely sequence of state transitions that may have led from an observed sequence of features (Pober et al., 2006; Ward et al., 2006; Lester et al., 2006; Lester et al., 2005).

#### 5.9.2 Application and limitations

HMMs have been employed in sedentary behavior classification both as standalone models and within multi-stage classification pipelines. They have been used to recognize sequences of movement and transitions between postures, enabling insight into the dynamics of inactivity. In contrast, simple Markov chains have been applied in cases where only transition probabilities between activities are needed, useful in analyzing periods of prolonged sedentary behavior (Preece et al., 2009; Krause et al., 2012). Despite their strengths in temporal modeling, HMMs have limitations such as the need for large, annotated datasets, difficulty in parameter estimation, and high computational cost when scaled to complex activities or multi-sensor inputs. While traditional probabilistic models like HMMs offer interpretability, recent deep learning architectures such as DeepSense have emerged as powerful alternatives for processing time-series data from wearable sensors. These frameworks combine convolutional and recurrent layers to capture both spatial and temporal features, improving classification accuracy in human activity recognition tasks (Yao et al., 2017).

### 5.10 Combining classifiers

#### 5.10.1 Mechanism

Classifier fusion—also known as ensemble learning—combines the outputs of multiple base classifiers to improve overall performance (Preece et al., 2009). Techniques such as bagging, boosting, and stacking can be used to generate meta-level predictions that often outperform any single method (Webb, 2002; Theodoridis and Koutroumbas, 2006; Ravi et al., 2005). These ensembles can combine classifiers of the same type or mix heterogeneous models like decision trees, SVMs, and kNN.

#### 5.10.2 Application and limitations

A pilot study by Ravi et al. (2005) tested a meta-classification system on data from two subjects performing eight activities. Five base-level classifiers—decision trees, decision tables, kNN, SVM, and Naïve Bayes—were compared to boosted and stacked ensemble methods (Ravi et al., 2005). The study found that boosted SVMs achieved the highest accuracy across different data partitions (Ravi et al., 2005). While ensemble approaches can enhance predictive accuracy and generalizability, they come at the cost of increased complexity and computational demand, making real-time implementation more challenging on resource-limited devices.

### 5.11 Unsupervised learning

#### 5.11.1 Mechanism

Unsupervised learning does not involve any activity labels for each data window. It is used for the identification of clusters of related patterns in the feature space (Duda et al., 2000; Theodoridis and Koutroumbas, 2006).

#### 5.11.2 Application and limitations

The benefit such techniques bring is that exploratory data analysis and investigation of the significance of individual features can be done (Preece et al., 2009; Duda et al., 2000; Theodoridis and Koutroumbas, 2006), which makes it much more specific in terms of analyzing prolonged physical inactivity. The unsupervised approach can also be combined with the supervised approach; consequently, off-the-shelf systems can be developed that can be trained by the user with periodic input (Preece et al., 2009). As a result, considerable flexibility and acquaintance with new scenarios faced by the real-world user can be accomplished (Preece et al., 2009). Moreover, unsupervised learning can be used as the initial stage of a system for detecting adverse events that are different from typical daily activity patterns (Preece et al., 2009).

In sedentary behavior research, unsupervised learning has been used to discover new activity patterns or identify anomalies such as unusual postural transitions. It also enables flexible systems that adapt to new environments by learning from user-provided feedback over time. One study demonstrated that unsupervised methods could serve as a first-stage classifier, flagging deviations from normal activity that may warrant further analysis (Van Laerhoven and Cakmakci, 2000). Although useful for personalization and novelty detection, these methods lack direct interpretability and may struggle to assign semantic meaning to discovered clusters without some level of supervision or contextual knowledge.

## 6 Summary of machine learning techniques

As technology has advanced over the years, machine learning has played a pivotal role in issues related to public health. Machine learning has been extensively used to study sedentary lifestyle and the health issues linked with it. Numerous machine learning algorithms that include k nearest neighbor (kNN), artificial neural networks (ANN), decision trees, Markov models, fuzzy logic, and support vector machines (SVM) have been utilized for the classification of physical activity; after comparing the performance characteristics of 11 different machine learning approaches mentioned above (Table 2), Preece et al. (2009) concluded that no classifier stands out as the best for a given activity classification problem. No single algorithm can be deemed the best given the fact that different classifiers are best for different problems. With many techniques being used on a small number of subjects, the need of the time is that future studies include a higher number of subjects (Preece et al., 2009).

**Table 2:** Machine learning techniques and their corresponding principles.

Machine learning technique	Principle
Threshold-based classification	Derived feature compared with the threshold that is determined beforehand in order to classify the activity
Hierarchical methods	Binary decision structure based on input features
Decision trees	Set of rules formed based on the discriminatory ability of the features one by one
k-nearest neighbor	Multi-dimensional feature space formed; k-nearest points of training data identified and majority of k-nearest neighbors corresponding to a specific activity determine the classification
Artificial neural networks	Different features constitute the input while different classes of activity represent the output
Support vector machines	Based on extracting optimal separating decision hyperplanes between classes with the maximum margin between patterns of each class
Naïve Bayes and Gaussian mixture models	Bayesian approach based on estimated conditional probabilities of signal patterns available from each activity class, and the input features considered independent of each other; Gaussian similar except that the likelihood function assumed to be of unknown shape and functional form
Fuzzy logic	Each input assigned the membership of a fuzzy class; application of rules (if-then statements) then produce a corresponding output
Markov chains and hidden Markov models	Each activity represented as a different state; can be used to represent the likelihood of transitions between different activities
Combining classifiers	Based on combining output using different techniques
Unsupervised learning	Involves no activity labels for each data window; used for the identification of clusters of related patterns in the feature space

## 7 Clinical predictive performance

### 7.1 Precision of machine learning in predicting sedentary behaviors

Machine learning has turned out to be a powerful tool to transform motion signals from wearable devices into different variables like postures, sedentary behaviors, and sleep (de Almeida et al., 2018). Machine learning techniques have resulted in more accurate measurement and classification of more sophisticated movements and postures from wearables (Farrahi et al., 2019). A systematic review focusing on the accuracy of machine learning models for activity prediction stated that these algorithms have the capacity to predict activity types accurately, irrespective of the site where accelerometer-based activity monitor is placed on the body (Farrahi et al., 2019). Ensemble methods that combine several ML algorithms have also shown promise in this field (Chowdhury et al., 2017). Moreover, the evolution of deep learning techniques—such as convolutional neural networks (CNNs) and recurrent neural networks (RNNs)—has significantly advanced time-series classification in human activity recognition (Ismail Fawaz et al., 2019), highlighting the potential of such methods for more refined predictions in sedentary behavior analysis.

Machine learning analysis of wearable sensors during daily life activities can help recognize sedentary behaviors (Kańtoch, 2018). One study worked on recognizing sedentary behavior by using the combination of data from smart shirt, individual factors, and machine learning algorithms (Kańtoch, 2018). The goal was to develop the method of automatic recognition of sedentary behavior with respect to cardiovascular risk by quantitative measurement of physical activity; for that, the idea was to design prototype of a smart shirt equipped with a processor, wearable sensors, power supply and telemedical interface (Kańtoch, 2018). The results showed that the method proposed could quantitatively measure sedentary behavior without restricting daily activities; the experimental results also evinced that machine learning approach can estimate sedentary behavior with high accuracy (Kańtoch, 2018). Reported accuracy rates across studies ranged from 84 to 92.4% for ML-based activity classification. For instance, Zhang et al. (2006) achieved 92.4% accuracy using an SVM model for fall detection. Decision trees in Bao and Intille’s (2004) study reached 86% accuracy in classifying 20 physical activities. Ensemble models, such as boosted SVM and CNN-based approaches, demonstrated superior prediction performance and reduced false positives in next-day sedentary behavior forecasting.

In another study, steps counting-based machine learning approach was used for the prediction of sedentary behavior (Papathomas et al., 2021). The objective was to analyze historical data from numerous individuals who used wearable physical activity trackers and explore the performance of four machine learning algorithms, namely logistic regression, random forest, XGBoost, and convolutional neural networks, and a majority vote ensemble of the algorithms (Papathomas et al., 2021). The results revealed that all models could effectively predict the next-day sedentary behavior; however, the ensemble model proved to be the best since it was more effective in predicting sedentary behavior and reducing false positives (Papathomas et al., 2021). Therefore, in designing appropriate interventions to tackle the issue of sedentary behavior, it can play a pivotal role.

With that, the increasing popularity of causal machine learning (CML) holds promise in the studies investigating health-related behaviors as it explores the reason why some people engage in healthier behaviors (Farrahi and Rostami, 2024). CML has the capability of investigating how a system would react to an intervention, meaning that the outcome can be predicted based on the treatment. When the effects of interventions can be quantified, actionable decisions can be made while maintaining robustness when confounders (variables that impact both dependent and independent variables) are present (Sanchez et al., 2022)—this adds to the effectiveness of machine learning in exploring health-related behaviors. Furthermore, with the widespread availability of open-source languages like Python and R, the application of machine learning by coding has become more accessible than ever (Raschka et al., 2020). Moreover, explainable artificial intelligence (XAI) is an active field of research (Loh et al., 2022). It is a technique which can provide confidence in the model’s prediction since it focuses on how the prediction is derived, which encourages the use of AI systems in healthcare (Loh et al., 2022). Therefore, machine learning can prove to be a useful tool to predict sedentary behavior.

## 8 Challenges and future direction

Despite the high potential of machine learning approach in studying sedentary lifestyle, there are some challenges and limitations that need to be addressed. For the analysis of sedentary behavior, the most commonly used wearable devices are activPAL, ActiGraph, and Active style Pro; instead of being integrated with clothes, these devices attach to the body using elastic belts leading to different outputs making it difficult to compare them (Sasai, 2017). Additionally, in most cases, when wearable devices are used to analyze sedentary behavior, the contextual information cannot be detected, and the computation is time-consuming (Sasai, 2017). Specifically, with regards to the use of smartwatches, the battery life, cost and optimizing hardware tend to be the fetters (Rawassizadeh et al., 2014). With that, while wearing a smartwatch, the user has higher motivation for an active lifestyle (Rawassizadeh et al., 2014). Furthermore, there is overrepresentation of wearable-based studies, and recent evidence suggests that machine learning models trained on such data may perpetuate algorithmic bias and health disparities, particularly among underrepresented populations. With respect to the methods used for sedentary behavior analysis, while ensemble methods that combine several ML algorithms have shown promise, to further advance the research in this field, machine learning algorithms need to be tested under fully free-living conditions (Farrahi and Rostami, 2024). With that, the future studies should focus on assessing the generalization performance of machine learning algorithms on datasets distinct from the ones used for their training (Farrahi and Rostami, 2024).

In addition to these technical challenges, several broader limitations and potential biases should be acknowledged. The scope of this review was limited to studies indexed in PubMed and Scopus, which may have excluded relevant literature from other databases or grey literature sources. This selection criterion could introduce selection bias. Furthermore, the reliance on published, peer-reviewed articles raises the possibility of publication bias, as studies with positive or significant findings are more likely to be reported. Another limitation stems from the overrepresentation of studies using wearable devices, which may not reflect populations in resource-limited settings. Many of the machine learning models discussed were trained on small datasets or tested in controlled environments, reducing their generalizability to real-world conditions. Additionally, there is inconsistency in how sedentary behavior is defined and measured across studies, making synthesis of results more challenging. Furthermore, socioeconomic factors play a crucial role in shaping sedentary behavior patterns, particularly in low-income populations who often face greater environmental and occupational constraints to physical activity (Owen et al., 2011). Lastly, several reviewed studies did not fully account for confounding variables, such as age or socioeconomic status, which could influence both sedentary behavior and health outcomes. Certain populations such as children, older adults, and individuals from diverse socioeconomic or cultural backgrounds were underrepresented in the reviewed studies, which limits the generalizability of the findings across age groups and communities. These limitations highlight the need for future studies to adopt more comprehensive and diverse methodologies.

## 9 Ethical considerations

ML applications for sedentary behavior monitoring through wearable technologies raise important ethical issues that warrant careful attention. These include concerns related to data privacy, algorithmic bias, and the need for explainable AI in clinical decision-making.

First, continuous data collection by wearables generates highly granular and sensitive information, such as movement patterns, heart rate, and location, which could lead to privacy breaches if not properly secured. Studies have found that many wearable device users are unaware of how their data is stored or shared. To address this, data encryption, anonymization, and informed consent protocols should be standardized. Second, algorithmic bias presents a substantial risk. ML models trained on non-representative datasets may perform poorly for underrepresented populations, such as the elderly, disabled individuals, or racial minorities. Lastly, the lack of model transparency in many ML systems limits their adoption in clinical settings. “Black-box” models may offer high predictive accuracy, but without explainability, clinicians may be hesitant to trust or act on ML recommendations. Explainable AI tools help demystify model predictions and foster accountability.

## 10 Conclusion

As physical inactivity and sedentary lifestyle have become “the disease” of the 21st century, there is an exigent need to tackle it. For that, over the years, research studies have focused on several sedentary behaviors and how they correlate with different health problems including metabolic diseases, cancer, musculoskeletal diseases, and depression. While previous research has heavily utilized different approaches of machine learning to explore physical inactivity and sedentary lifestyle, they have also evidenced the effectiveness of distinct machine learning algorithms. That signifies how useful machine learning has been in studying sedentary behaviors. Considering the significant impact of sedentary lifestyles on health, there is an urgent need to expand research by including larger, more diverse populations and applying various machine learning algorithms under real-life conditions. Leveraging big data from wearable devices and other digital sources can enhance the precision and applicability of these algorithms, enabling deeper insights into sedentary behavior patterns. To address this issue on a global scale, developed nations should lead by establishing comprehensive data-sharing initiatives and integrating big data analytics into public health strategies. Such collaborative efforts would support the development of effective, evidence-based interventions and encourage other countries to adopt similar approaches, ultimately reducing the global burden of non-communicable diseases linked to inactivity.

## References

[ref1] Allen F. R. Ambikairajah E. Lovell N. H. Celler B. G. (2006). Classification of a known sequence of motions and postures from accelerometry data using adapted Gaussian mixture models. Physiol. Meas. 27, 935–951. doi: 10.1088/0967-3334/27/10/001, PMID: 16951454

[ref2] Aminian K. Robert P. Jéquier E. Schutz Y. (1995). Incline, speed, and distance assessment during unconstrained walking. Med. Sci. Sports Exerc. 27, 226–234. doi: 10.1249/00005768-199502000-00012, PMID: 7723646

[ref3] An H. Y. Chen W. Wang C. W. Yang H. F. Huang W. T. Fan S. Y. (2020). The relationships between physical activity and life satisfaction and happiness among young, middle-aged, and older adults. Int. J. Environ. Res. Public Health 17:4817. doi: 10.3390/ijerph17134817, PMID: 32635457 PMC7369812

[ref4] Bao L. Intille S. (2004). “Activity recognition from user-annotated acceleration data” in Pervasive computing (Berlin: Springer), 1–17.

[ref5] Bellazzi R. Ferrazzi F. Sacchi L. (2011). Predictive data mining in clinical medicine: a focus on selected methods and applications. Wiley Interdiscip. Rev. Data Mining Knowl. Discov. 1, 416–430. doi: 10.1002/widm.23

[ref6] Biswas A. Chen C. Dobson K. G. Prince S. A. Shahidi F. V. Smith P. M.. (2023). Identifying the sociodemographic and work-related factors related to workers’ daily physical activity using a decision tree approach. BMC Public Health 23:1853. doi: 10.1186/s12889-023-16747-9, PMID: 37741965 PMC10517528

[ref7] Biswas A. Oh P. I. Faulkner G. E. Bajaj R. R. Silver M. A. Mitchell M. S.. (2015). Sedentary time and its association with risk for disease incidence, mortality, and hospitalization in adults. Ann. Intern. Med. 162, 123–132. doi: 10.7326/M14-1651, PMID: 25599350

[ref8] Bourke A. K. Lyons G. M. (2008). A threshold-based fall-detection algorithm using a bi-axial gyroscope sensor. Med. Eng. Phys. 30, 84–90. doi: 10.1016/j.medengphy.2006.12.001, PMID: 17222579

[ref9] Boyle J. Karunanithi M. Wark T. Chan W. Colavitti C. (2006). Quantifying functional mobility progress for chronic disease management. 2006 International Conference of the IEEE Engineering in Medicine and Biology Society. 10.1109/IEMBS.2006.260426 17945919

[ref10] Buck C. Loyen A. Foraita R. Van Cauwenberg J. De Craemer M. Mac Donncha C.. (2019). Factors influencing sedentary behaviour: a system based analysis using Bayesian networks within DEDIPAC. PLoS One 14:e0211546. doi: 10.1371/journal.pone.0211546, PMID: 30699199 PMC6353197

[ref11] Bussmann J. B. Martens W. L. Tulen J. H. Schasfoort F. C. van den Berg-Emons H. J. Stam H. J. (2001). Measuring daily behavior using ambulatory accelerometry: the activity monitor. Behav. Res. Methods Instrum. Comput. 33, 349–356. doi: 10.3758/bf03195388, PMID: 11591066

[ref12] Chastin S. F. M. Mandrichenko O. Helbostadt J. L. Skelton D. A. (2014). Associations between objectively-measured sedentary behaviour and physical activity with bone mineral density in adults and older adults, the NHANES study. Bone 64, 254–262. doi: 10.1016/j.bone.2014.04.009, PMID: 24735973

[ref13] Chen J. Kwong K. Chang D. Luk J. Bajcsy R. (2005). Wearable sensors for reliable fall detection. 2005 IEEE Engineering in Medicine and Biology 27th Annual Conference. 3551–3554. 10.1109/IEMBS.2005.1617246 17280991

[ref14] Cheng L. De Vos J. Zhao P. Yang M. Witlox F. (2020). Examining non-linear built environment effects on elderly’s walking: a random forest approach. Transp. Res. D 88:102552. doi: 10.1016/j.trd.2020.102552

[ref15] Chowdhury A. K. Tjondronegoro D. Chandran V. Trost S. G. (2017). Ensemble methods for classification of physical activities from wrist accelerometry. Med. Sci. Sports Exerc. 49, 1965–1973. doi: 10.1249/MSS.0000000000001291, PMID: 28419025

[ref16] Coley B. Najafi B. Paraschiv-Ionescu A. Aminian K. (2005). Stair climbing detection during daily physical activity using a miniature gyroscope. Gait Posture 22, 287–294. doi: 10.1016/j.gaitpost.2004.08.008, PMID: 16274909

[ref17] Cristianini N. Shawe-Taylor J. (2001). An introduction to support vector machines and other kernel-based learning methods. Cambridge: Cambridge University Press.

[ref18] Culhane K. M. Lyons G. M. Hilton D. Grace P. A. Lyons D. (2004). Long-term mobility monitoring of older adults using accelerometers in a clinical environment. Clin. Rehabil. 18, 335–343. doi: 10.1191/0269215504cr734oa, PMID: 15137565

[ref19] de Almeida M. M. da Silva I. C. M. Ramires V. V. Reichert F. F. Martins R. C. Tomasi E. (2018). Calibration of raw accelerometer data to measure physical activity: a systematic review. Gait Posture 61, 98–110. doi: 10.1016/j.gaitpost.2017.12.028 29324298

[ref20] Dempsey P. C. Larsen R. N. Dunstan D. W. Owen N. Kingwell B. A. (2018). Sitting less and moving more. Hypertension 72, 1037–1046. doi: 10.1161/HYPERTENSIONAHA.118.11190, PMID: 30354827 PMC7343526

[ref21] Duda R. O. Hart P. E. Stork D. G. (2000). Pattern classification. 2nd Edn. Hoboken, NJ: John Wiley & Sons, Ltd., 688.

[ref22] Farrahi V. Niemelä M. Kangas M. Korpelainen R. Jämsä T. (2019). Calibration and validation of accelerometer-based activity monitors: a systematic review of machine-learning approaches. Gait Posture 68, 285–299. doi: 10.1016/j.gaitpost.2018.12.003, PMID: 30579037

[ref23] Farrahi V. Rostami M. (2024). Machine learning in physical activity, sedentary, and sleep behavior research. J. Act. Sedent. Sleep Behav. 3:5. doi: 10.1186/s44167-024-00045-9, PMID: 40217437 PMC11960357

[ref24] Fennell C. Barkley J. E. Lepp A. (2019). The relationship between cell phone use, physical activity, and sedentary behavior in adults aged 18–80. Comput. Human Behav. 90, 53–59. doi: 10.1016/j.chb.2018.08.044

[ref25] Freedson P. Bowles H. R. Troiano R. Haskell W. (2012). Assessment of physical activity using wearable monitors: recommendations for monitor calibration and use in the field. Med. Sci. Sports Exerc. 44:S1. doi: 10.1249/MSS.0b013e3182399b7e, PMID: 22157769 PMC3245520

[ref26] Goulermas J. Y. Findlow A. H. Nester C. J. Liatsis P. Zeng X. J. Kenney L. P.. (2008). An instance-based algorithm with auxiliary similarity information for the estimation of gait kinematics from wearable sensors. IEEE Trans. Neural Netw. 19, 1574–1582. doi: 10.1109/TNN.2008.2000808, PMID: 18779089

[ref27] Goulermas J. Y. Howard D. Nester C. J. Jones R. K. Ren L. (2005). Regression techniques for the prediction of lower limb kinematics. J. Biomech. Eng. 127, 1020–1024. doi: 10.1115/1.2049328, PMID: 16438243

[ref28] Greenwood-Hickman M. A. Nakandala S. Jankowska M. M. Rosenberg D. E. Tuz-Zahra F. Bellettiere J.. (2021). The CNN hip accelerometer posture (CHAP) method for classifying sitting patterns from hip accelerometers: a validation study. Med. Sci. Sports Exerc. 53, 2445–2454. doi: 10.1249/MSS.0000000000002705, PMID: 34033622 PMC8516667

[ref29] Hamilton M. T. Hamilton D. G. Zderic T. W. (2007). Role of low energy expenditure and sitting in obesity, metabolic syndrome, type 2 diabetes, and cardiovascular disease. Diabetes 56, 2655–2667. doi: 10.2337/db07-0882, PMID: 17827399

[ref30] Hamilton M. T. Healy G. N. Dunstan D. W. Zderic T. W. Owen N. (2008). Too little exercise and too much sitting: inactivity physiology and the need for new recommendations on sedentary behavior. Curr. Cardiovasc. Risk Rep. 2, 292–298. doi: 10.1007/s12170-008-0054-8, PMID: 22905272 PMC3419586

[ref31] Haug C. J. Drazen J. M. (2023). Artificial intelligence and machine learning in clinical medicine. N. Engl. J. Med. 388, 1201–1208. doi: 10.1056/NEJMra2302038 36988595

[ref32] Healy G. N. Wijndaele K. Dunstan D. W. Shaw J. E. Salmon J. Zimmet P. Z.. (2008). Objectively measured sedentary time, physical activity, and metabolic risk: the Australian diabetes, obesity and lifestyle study (Aus Diab). Diabetes Care 31, 369–371. doi: 10.2337/dc07-1795, PMID: 18000181

[ref33] Huang Y. Li L. Gan Y. Wang C. Jiang H. Cao S.. (2020). Sedentary behaviors and risk of depression: a meta-analysis of prospective studies. Transl. Psychiatry 10:26. doi: 10.1038/s41398-020-0715-z, PMID: 32066686 PMC7026102

[ref34] Hwang J. Y. Kang J. M. Jang Y. W. Kim H. (2004). Development of novel algorithm and real-time monitoring ambulatory system using Bluetooth module for fall detection in the elderly. The 26th Annual International Conference of the IEEE Engineering in Medicine and Biology Society. 2204–2207. 10.1109/IEMBS.2004.1403643 17272163

[ref35] Ismail Fawaz H. Forestier G. Weber J. Idoumghar L. Muller P.-A. (2019). Deep learning for time series classification: a review. Data Min. Knowl. Discov. 33, 917–963. doi: 10.1007/s10618-019-00619-1

[ref36] Javaid M. Haleem A. Singh R. P. Suman R. Rab S. (2022). Significance of machine learning in healthcare: features, pillars and applications. Int. J. Intell. Netw. 3, 58–73. doi: 10.1016/j.ijin.2022.05.002

[ref37] Jochem C. Wallmann-Sperlich B. Leitzmann M. F. (2019). The influence of sedentary behavior on cancer risk: epidemiologic evidence and potential molecular mechanisms. Curr. Nutr. Rep. 8, 167–174. doi: 10.1007/s13668-019-0263-4, PMID: 30887424

[ref38] Kańtoch E. (2018). Recognition of sedentary behavior by machine learning analysis of wearable sensors during activities of daily living for telemedical assessment of cardiovascular risk. Sensors 18:3219. doi: 10.3390/s18103219, PMID: 30249987 PMC6210891

[ref39] Karantonis D. M. Narayanan M. R. Mathie M. Lovell N. H. Celler B. G. (2006). Implementation of a real-time human movement classifier using a triaxial accelerometer for ambulatory monitoring. IEEE Trans. Inf. Technol. Biomed. 10, 156–167. doi: 10.1109/TITB.2005.856864, PMID: 16445260

[ref40] Katzmarzyk P. T. (2010). Physical activity, sedentary behavior, and health: paradigm paralysis or paradigm shift? Diabetes 59, 2717–2725. doi: 10.2337/db10-0822, PMID: 20980470 PMC2963526

[ref41] Katzmarzyk P. T. Powell K. E. Jakicic J. M. Troiano R. P. Piercy K. Tennant B. (2019). Sedentary behavior and health: update from the 2018 physical activity guidelines advisory committee. Med. Sci. Sports Exerc. 51, 1227–1241. doi: 10.1249/MSS.0000000000001935, PMID: 31095080 PMC6527341

[ref42] Krause A. Ihmig M. Rankin E. Leong D. Gupta S. Siewiorek D. (2012). Trading off prediction accuracy and power consumption for context-aware wearable computing: 16th International Symposium on Wearable Computers

[ref43] Laerhoven K. V. Gellersen H. W. (2004). Spine versus porcupine: a study in distributed wearable activity recognition. Eighth International Symposium on Wearable Computers.

[ref44] Lee S.-H. Son C. Yeo S. Ha I.-H. (2019). Cross-sectional analysis of self-reported sedentary behaviors and chronic knee pain among South Korean adults over 50 years of age in KNHANES 2013–2015. BMC Public Health 19:1375. doi: 10.1186/s12889-019-7653-9, PMID: 31655569 PMC6815384

[ref45] Lester J. Choudhury T. Borriello G. (Eds.) (2006). “A practical approach to recognizing physical activities” in Pervasive computing (Berlin: Springer).

[ref46] Lester J. Choudhury T. Kern N. Borriello G. Hannaford B. (2005). A hybrid discriminative/generative approach for modeling human activities. Proceedings of the Nineteenth International Joint Conference on Artificial Intelligence. 766–772

[ref47] Lindemann U. Hock A. Stuber M. Keck W. Becker C. (2005). Evaluation of a fall detector based on accelerometers: a pilot study. Med. Biol. Eng. Comput. 43, 548–551. doi: 10.1007/BF02351026, PMID: 16411625

[ref48] Liu M. Wu L. Yao S. (2016). Dose-response association of screen time-based sedentary behaviour in children and adolescents and depression: a meta-analysis of observational studies. Br. J. Sports Med. 50, 1252–1258. doi: 10.1136/bjsports-2015-095084, PMID: 26552416 PMC4977203

[ref49] Loh H. W. Ooi C. P. Seoni S. Barua P. D. Molinari F. Acharya U. R. (2022). Application of explainable artificial intelligence for healthcare: a systematic review of the last decade (2011–2022). Comput. Methods Prog. Biomed. 226:107161. doi: 10.1016/j.cmpb.2022.107161, PMID: 36228495

[ref50] Lynch B. M. (2010). Sedentary behavior and cancer: a systematic review of the literature and proposed biological mechanisms. Cancer Epidemiol. Biomarkers Prev. 19, 2691–2709. doi: 10.1158/1055-9965.EPI-10-0815, PMID: 20833969

[ref51] Maurer U. Rowe A. Smailagic A. Siewiorek D. (2006). “Location and activity recognition using eWatch: a wearable sensor platform” in Ambient intelligence in everyday life: foreword by Emile Aarts. eds. Cai Y. Abascal J. (Berlin: Springer), 86–102.

[ref52] Najafi B. Aminian K. Paraschiv-Ionescu A. Loew F. Büla C. J. Robert P. (2003). Ambulatory system for human motion analysis using a kinematic sensor: monitoring of daily physical activity in the elderly. IEEE Trans. Biomed. Eng. 50, 711–723. doi: 10.1109/TBME.2003.812189, PMID: 12814238

[ref53] Narayanan A. Desai F. Stewart T. Duncan S. Mackay L. (2020). Application of raw accelerometer data and machine-learning techniques to characterize human movement behavior: a systematic scoping review. J. Phys. Act. Health 17, 360–383. doi: 10.1123/jpah.2019-0088, PMID: 32035416

[ref54] Newton R. L. Jr. Han H. Zderic T. Hamilton M. (2013). The energy expenditure of sedentary behavior: a whole room calorimeter study. PLoS One 8:e63171. doi: 10.1371/journal.pone.0063171, PMID: 23658805 PMC3643905

[ref55] Ohlsson C. Gidestrand E. Bellman J. Larsson C. Palsdottir V. Hägg D.. (2020). Increased weight loading reduces body weight and body fat in obese subjects—a proof of concept randomized clinical trial. EClinicalMedicine 22:100338. doi: 10.1016/j.eclinm.2020.100338, PMID: 32510046 PMC7264953

[ref56] Owen N. (2012). Sedentary behavior: understanding and influencing adults’ prolonged sitting time. Prev. Med. 55, 535–539. doi: 10.1016/j.ypmed.2012.08.024, PMID: 22968124

[ref57] Owen N. Sugiyama T. Eakin E. E. Gardiner P. A. Tremblay M. S. Sallis J. F. (2011). Adults’ sedentary behavior determinants and interventions. Am. J. Prev. Med. 41, 189–196. doi: 10.1016/j.amepre.2011.05.013, PMID: 21767727

[ref58] Papathomas E. Triantafyllidis A. Mastoras R. E. Giakoumis D. Votis K. Tzovaras D. (2021). A machine learning approach for prediction of sedentary behavior based on daily step counts. 2021 43rd Annual International Conference of the IEEE Engineering in Medicine & Biology Society (EMBC) 10.1109/EMBC46164.2021.9630894 34891316

[ref59] Park J. H. Moon J. H. Kim H. J. Kong M. H. Oh Y. H. (2020). Sedentary lifestyle: overview of updated evidence of potential health risks. Korean J. Fam. Med. 41, 365–373. doi: 10.4082/kjfm.20.0165, PMID: 33242381 PMC7700832

[ref60] Parkka J. Ermes M. Korpipaa P. Mantyjarvi J. Peltola J. Korhonen I. (2006). Activity classification using realistic data from wearable sensors. IEEE Trans. Inf. Technol. Biomed. 10, 119–128. doi: 10.1109/TITB.2005.856863, PMID: 16445257

[ref61] Pober D. M. Staudenmayer J. Raphael C. Freedson P. S. (2006). Development of novel techniques to classify physical activity mode using accelerometers. Med. Sci. Sports Exerc. 38, 1626–1634. doi: 10.1249/01.mss.0000227542.43669.45, PMID: 16960524

[ref62] Preece S. J. Goulermas J. Y. Kenney L. P. Howard D. Meijer K. Crompton R. (2009). Activity identification using body-mounted sensors—a review of classification techniques. Physiol. Meas. 30, R1–R33. doi: 10.1088/0967-3334/30/4/R01 19342767

[ref63] Rajpurkar P. Chen E. Banerjee O. Topol E. J. (2022). AI in health and medicine. Nat. Med. 28, 31–38. doi: 10.1038/s41591-021-01614-0, PMID: 35058619

[ref64] Raschka S. Patterson J. Nolet C. (2020). Machine learning in Python: main developments and technology trends in data science, machine learning, and artificial intelligence. Information 11:193. doi: 10.3390/info11040193

[ref65] Ravi N. Dandekar N. Mysore P. Littman M. (2005). Activity recognition from accelerometer data. The 20th National Conference on Artificial Intelligence and the 17th Innovative Applications of Artificial Intelligence Conference. 1541–1546.

[ref66] Rawassizadeh R. Price B. A. Petre M. (2014). Wearables: has the age of smartwatches finally arrived? Commun. ACM 58, 45–47. doi: 10.1145/2629633

[ref67] Saint-Maurice P. F. Kim Y. Welk G. J. Gaesser G. A. (2016). Kids are not little adults: what MET threshold captures sedentary behavior in children? Eur. J. Appl. Physiol. 116, 29–38. doi: 10.1007/s00421-015-3238-1, PMID: 26271677

[ref68] Sanchez P. Voisey J. P. Xia T. Watson H. I. O’Neil A. Q. Tsaftaris S. A. (2022). Causal machine learning for healthcare and precision medicine. R. Soc. Open Sci. 9:220638. doi: 10.1098/rsos.220638, PMID: 35950198 PMC9346354

[ref69] Sasai H. (2017). Assessing sedentary behavior using wearable devices: an overview and future directions. J. Phys. Fitness Sports Med. 6, 135–143. doi: 10.7600/jpfsm.6.135

[ref70] Schmid D. Leitzmann M. F. (2014). Television viewing and time spent sedentary in relation to cancer risk: a meta-analysis. JNCI J. Natl. Cancer Inst. 106:dju098. doi: 10.1093/jnci/dju098, PMID: 24935969

[ref71] Teychenne M. Costigan S. A. Parker K. (2015). The association between sedentary behaviour and risk of anxiety: a systematic review. BMC Public Health 15:513. doi: 10.1186/s12889-015-1843-x, PMID: 26088005 PMC4474345

[ref72] Theodoridis S. Koutroumbas K. (2006). Pattern recognition. 3rd Edn. London: Academic Press.

[ref73] Tworoger S. S. Missmer S. A. Eliassen A. H. Barbieri R. L. Dowsett M. Hankinson S. E. (2007). Physical activity and inactivity in relation to sex hormone, prolactin, and insulin-like growth factor concentrations in premenopausal women. Cancer Causes Control 18, 743–752. doi: 10.1007/s10552-007-9017-5, PMID: 17549594

[ref74] Van Laerhoven K. Cakmakci O. (2000). What shall we teach our pants?. Fourth International Symposium on Wearable Computers. 77–83

[ref75] Vapnik V. N. (1998). Statistical learning theory. Chichester: Wiley.

[ref76] Ward J. A. Lukowicz P. Troster G. Starner T. E. (2006). Activity recognition of assembly tasks using body-worn microphones and accelerometers. IEEE Trans. Pattern Anal. Mach. Intell. 28, 1553–1567. doi: 10.1109/TPAMI.2006.197 16986539

[ref77] Waters C. N. Ling E. P. Chu A. H. Y. Ng S. H. X. Chia A. Lim Y. W.. (2016). Assessing and understanding sedentary behaviour in office-based working adults: a mixed-method approach. BMC Public Health 16:360. doi: 10.1186/s12889-016-3023-z, PMID: 27117178 PMC4847225

[ref78] Webb A. R. (2002). Statistical pattern recognition. Hoboken, NJ: John Wiley & Sons, Ltd.

[ref79] WHO (2024). Nearly 1.8 billion adults at risk of disease from not doing enough physical activity. Available online at: https://www.who.int/news/item/26-06-2024-nearly-1.8-billion-adults-at-risk-of-disease-from-not-doing-enough-physical-activity (Accessed November 6, 2024). PMC11288492 39074895

[ref80] Wiemken T. L. Kelley R. R. (2020). Machine learning in epidemiology and health outcomes research. Annu. Rev. Public Health 41, 21–36. doi: 10.1146/annurev-publhealth-040119-094437, PMID: 31577910

[ref81] World Health Organization (2020). Physical inactivity: a global public health problem. Geneva: World Health Organization. Available online at: https://www.who.int/dietphysicalactivity/factsheet_inactivity/en/

[ref82] Yao S. Hu S. Zhao Y. Zhang A. Abdelzaher T.. (2017). Deep sense: a unified deep learning framework for time-series mobile sensing data processing. Proceedings of the 26th International Conference on World Wide Web. 351–360.

[ref83] Zhang T. Wang J. Liu P. Hou J. (2005). Fall detection by embedding an accelerometer in cellphone and using KFD algorithm. Int. J. Comput. Sci. Netw. Secur. 6, 277–284.

[ref84] Zhang T. Wang J. Xu L. Liu P. (2006). “Using wearable sensor and NMF algorithm to realize ambulatory fall detection” in Advances in natural computation (Berlin: Springer).

[ref85] Zhou J. Zhou Q. Wang D. P. Zhang T. Wang H. J. Song Y.. (2017). Associations of sedentary behavior and physical activity with dyslipidemia. Beijing Da Xue Xue Bao 49, 418–423. 28628141

